# Incidence of catheter-related complications in patients with central venous or hemodialysis catheters: a health care claims database analysis

**DOI:** 10.1186/1471-2261-13-86

**Published:** 2013-10-16

**Authors:** Pavel Napalkov, Diana M Felici, Laura K Chu, Joan R Jacobs, Susan M Begelman

**Affiliations:** 1 Genentech, Inc., 1 DNA Way, South San Francisco, CA 94080, USA

**Keywords:** Adverse events, Bloodstream infections, Catheters, Catheter-related complication, Catheter-related thrombosis

## Abstract

**Background:**

Central venous catheter (CVC) and hemodialysis (HD) catheter usage are associated with complications that occur during catheter insertion, dwell period, and removal. This study aims to identify and describe the incidence rates of catheter-related complications in a large patient population in a United States-based health care claims database after CVC or HD catheter placement.

**Methods:**

Patients in the i3 InVision DataMart^®^ health care claims database with at least 1 CVC or HD catheter insertion claim were categorized into CVC or HD cohorts using diagnostic and procedural codes from the US Renal Data System, American College of Surgeons, and American Medical Association’s Physician Performance Measures. Catheter-related complications were identified using published diagnostic and procedural codes. Incidence rates (IRs)/1000 catheter-days were calculated for complications including catheter-related bloodstream infections (CRBSIs), thrombosis, embolism, intracranial hemorrhage (ICH), major bleeding (MB), and mechanical catheter–related complications (MCRCs).

**Results:**

Thirty percent of the CVC cohort and 54% of the HD cohort had catheter placements lasting <90 days. Catheter-related complications occurred most often during the first 90 days of catheter placement. IRs were highest for CRBSIs in both cohorts (4.0 [95% CI, 3.7-4.3] and 5.1 [95% CI, 4.7-5.6], respectively). Other IRs in CVC and HD cohorts, respectively, were thrombosis, 1.3 and 0.8; MCRCs, 0.6 and 0.7; embolism, 0.4 and 0.5; MB, 0.1 and 0.3; and ICH, 0.1 in both cohorts. Patients with cancer at baseline had significantly higher IRs for CRBSIs and thrombosis than non-cancer patients. CVC or HD catheter–related complications were most frequently seen in patients 16 years or younger.

**Conclusions:**

The risk of catheter-related complications is highest during the first 90 days of catheter placement in patients with CVCs and HD catheters and in younger patients (≤16 years of age) with HD catheters. Data provided in this study can be applied toward improving patient care.

## Background

Central venous catheters (CVCs) refer to prolonged vascular access devices indicated for the administration of intravenous medication treatments, fluids, or total parenteral nutrition, repeated blood sampling, and for hemodialysis (HD) [1,2]. Annual CVC exposure in hospital intensive care units has been estimated to total 15 million days [3] in the United States. Use of CVCs for HD (hereafter referred to as HD catheters) has increased in recent years, comprising approximately 25% of prevalent HD patients in the United States [4]; this is despite the recommendation by the National Kidney Foundation that tunneled, cuffed catheters for HD access be limited to <10% of prevalent dialysis patients due to the greater risk of morbidity and mortality [5]. Long-term dialysis using tunneled, cuffed catheters increases a patient’s risk of death 2- to 3-fold and serious infection 5- to 10-fold compared with dialysis using arteriovenous fistulas [1]. Additionally, compared with the general population, dialysis patients have a 100-fold greater risk of sepsis-related death, with infection-related and all-cause mortality highest in those with catheters [6].

CVC- and HD-catheter usage are associated with complications that occur during catheter insertion, throughout the catheter dwell period, and at the time of removal. Identification and prevention of catheter-related complications is critical to improving patient care [7,8]. Common complications include catheter misplacement or breakage, catheter occlusion due to local or systemic infection, and thrombosis [7-11].

Reported incidence rates (IRs) of catheter-related complications vary widely depending on the terminology and definition of complications, patient population, units of measurement, duration of catheterization and follow-up, catheter location, placement and care procedures, and diagnostic methods [9]. Patients undergoing HD may have different complication rates than non-HD patients. The US Renal Data System provides guidelines for the coding of HD catheter procedures [10-12]. Therefore, investigating complication rates in patients with and without HD catheter procedures is possible and important for understanding IRs in different patient groups.

The most common type of complication is catheter-related bloodstream infection (CRBSI), with an incidence rate of 0.46 to 30 per 1000 catheter-days, or in 4.3% to 26% of placed catheters [13-19]. In addition, many central venous access devices (CVADs) develop a fibrin layer (usually within 24 hours after insertion) which may cause CVAD dysfunction over time [20]. Catheter-related thrombosis has been reported in 0.6% to 33% of catheters, or 0.06 to 21 episodes/1000 catheter-days [9,14,21-23]. In patients with hematologic malignancy, the incidence of symptomatic CVC-related thrombosis varies between 1.2% and 13% [9]. Other less common complications that may be related to the treatment of occluded catheters include intracranial hemorrhage (ICH), major bleeding (MB), and embolism [21,24-26].

The objective of this study was to obtain unadjusted IRs of first complications after catheter insertion in a real-world setting. Previous studies have reported complication rates in patients with catheters; however, to our knowledge no studies have described incident complications of CVCs, including HD catheters, in both adults and children at different time periods following insertion. The current study uses a consistent methodologic approach to identify and describe the IRs of select first complication events after catheter insertion in a large, geographically diverse US patient cohort. This is important for improving patient care.

## Methods

### Study design and data source

A retrospective cohort analysis of the i3 InVision DataMart^®^ administrative claims database was conducted to determine the IRs of select complications in patients with either CVC or HD catheter replacement or removal. The i3 InVision database is a proprietary sample of individuals receiving health insurance benefits from a large health plan, comprising discounted fee-for-service independent practice association plans throughout the United States. The database includes medical, pharmacy, and limited laboratory claims for more than 39 million patients. Of these, over 24 million have been continuously enrolled for 12 or more months during the study period, representing approximately 8.2% of the 2000–2007 general US population [27].

Patients in the database between May 2000 and January 2007, with at least 1 claim for CVC or HD catheter insertion and a record of having a catheter removal or replacement procedure, were included in the initial data cut. Among these patients, those who were continuously enrolled in the health plan for 180 days prior to the first CVC or HD catheter placement and who had no complication events during this 180-day period were included in the study. Requiring patients to be free of defined complications prior to catheter placement and to have both catheter placement *and* removal or replacement claims allowed us to more accurately attribute study outcome events to catheter procedures in order to ensure a meaningful interpretation of catheter-related complications from claims data.

Patients were followed from the date of their first qualifying claim for insertion of either type of device (index date) until onset date of each distinct CVC or HD catheter–related complication event or other censoring date, such as the date of catheter removal or replacement, health plan termination, or end of study, whichever occurred first. In patients with multiple catheter insertion and removal or replacement claims, only the patient’s first qualifying placement claim was counted as the index date for IR calculation of study outcomes. In patients who were censored at catheter removal or replacement, only the patient’s first such claim was counted if multiple removal or replacement claims were on record.

Catheter placements, as well as removals and replacements were identified using combinations of codes from the International Classification of Diseases, Ninth Revision, Clinical Modification (ICD-9-CM) [28], Current Procedural Terminology (CPT), and Healthcare Common Procedure Coding System (HCPCS) based on categories and groupings proposed by the US Renal Data System [10-12], the American College of Surgeons [29], and the Physician Consortium for Performance Improvement [30]. Patients were categorized into 2 mutually exclusive cohorts (CVC or HD catheter, with HD catheter placement taking precedence given the greater specificity of claim codes consistent with HD catheter insertion). Patients with an HD catheter placement claim who also had at least 1 claim for chemotherapy or parenteral nutrition in the 30-day period prior to or following their HD catheter insertion claim were excluded. Patients in the CVC cohort were further classified as those with or without cancer at baseline (i.e., prior to CVC placement) using ICD-9-CM diagnosis codes for malignant neoplasms. Codes and definitions used to identify and assign patients into cohorts are listed in Additional file 1.

### Outcome events

Outcome events included first (incident) complications after catheter insertion that occurred during at least 1 overnight hospitalization or a hospital emergency room visit which did or did not result in hospitalization and were each identified using ICD-9-CM diagnostic or CPT procedure codes (Additional file 1) consistent with catheter-related blood stream infections, thrombosis, embolism, intracranial hemorrhage, major bleeding events, and mechanical catheter–related complications (MCRCs). The first or only occurrence of any of the above complications was also defined as an outcome and used for the IR calculation of the “Any complication” category. In patients with only 1 or multiple complications of the same type, the first or only complication occurrence was included in the IR calculation for specific types of events. In patients with multiple complication events of different types, the first occurrence of each distinct complication was used for the IR calculation of specific event types.

### Statistical analyses

Patient characteristics, including gender and age (with categories of <2, 2–16, 17–64, and ≥65 years) and duration of catheter placement (with time periods of 1 to <90, 90 to <180, 180 to <365, and ≥365 days) were reported for the HD and CVC cohorts as well as for the CVC patients by cancer-at-baseline status; the HD cohort did not include any patients younger than 2 years of age. IRs were calculated as the total number of complication events that occurred within the defined catheter placement period divided by the sum of the catheter-days at risk during the period of catheter placement and were expressed per 1000 catheter-days. The 95% confidence interval (CI) of the IR was calculated based on a normal approximation, under the assumption that the number of events follows a Poisson distribution. The 95% CI was expressed as: Exp{ln(incidence rate) ± 1.96 sqrt(1/total number of events)}. Catheter-days at risk were defined as the number of days between catheter insertion and event onset date (i.e., first outcome event) or the earliest date of catheter removal or replacement, health plan termination, or end of study. Catheter-days differed for each complication event and within each event catheter placement duration period because patients were censored at variable event dates. Fisher’s exact test was used to determine statistical significance of differences in IRs between CVC patients with or without cancer at baseline.

A sensitivity analysis was also conducted to determine if eliminating the requirement for having a catheter removal or replacement claim had an effect on complication rates. We calculated the IRs of complications in the first 90 days of catheter placement by using less stringent censoring criteria and eliminating the requirement to have a catheter removal or replacement claim.

## Results

### Patient disposition and characterization

**Figure 1:**
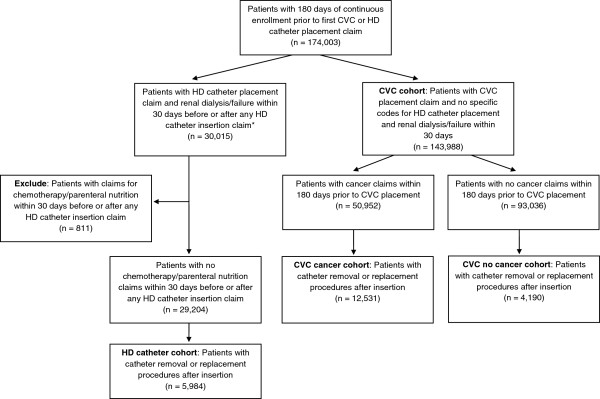
**Patient assignment into CVC and HD cohorts.** CPT, Current Procedural Terminology; CVC, central venous catheter; HD, hemodialysis. *With the exception of CPT code 36800 “Insertion of cannula for hemodialysis, other purpose (separate procedure); vein to vein” that does not require an accompanying renal dialysis or renal failure claim.

Patient assignment to the CVC and HD catheter cohorts is represented in Figure 1. Included in the study were 16,721 and 5,984 patients who underwent removal or replacement of a CVC or HD catheter, respectively. Patient characteristics, including duration of catheter placement are summarized in Table 1.

**Table 1:** Characteristics of patients with central venous and hemodialysis catheters^*^

**Characteristic**	**HD catheters**	**CVC all**	**CVC**	**CVC**
	**n = 5,984**	**n = 16,721**	**cancer**	**no cancer**
			**n = 12,531**	**n = 4,190**
Age, years, n (%)				
<2	0 (0)	188 (1.2)	66 (0.5)	122 (2.9)
2–16	115 (1.9)	933 (5.6)	449 (3.6)	484 (11.6)
17–64	4,052 (67.7)	13,655 (81.7)	10,665 (85.1)	2,990 (71.4)
≥65	1,817 (30.4)	1,945 (11.6)	1,351 (10.8)	594 (14.2)
Mean (SD)	56.1 (16.1)	48.1 (16.4)	49.5 (14.4)	43.9 (20.8)
Median (IQR)	57 (46, 68)	50 (41, 58)	51 (43, 58)	47 (32, 58)
Sex, n (%)				
Female	2,646 (44.2)	11,281 (67.5)	9,068 (72.4)	2,213 (52.9)
Male	3,338 (55.8)	5,440 (32.5)	3,463 (27.6)	1,977 (47.2)
Duration of catheter placement,^†^ days, n (%)				
1 to <90	3,213 (53.7)	5,060 (30.3)	2,479 (19.8)	2,581 (61.6)
90 to <180	1,344 (22.5)	4,029 (24.1)	3,498 (27.9)	531 (12.7)
180 to <365	932 (15.6)	4,811 (28.8)	4,286 (34.2)	525 (12.5)
≥365	495 (8.3)	2,821 (16.9)	2,268 (18.1)	553 (13.2)
Mean (SD)	145 (208.5)	219.5 (227.4)	238.8 (214.1)	161.8 (254.6)
Median (IQR)	78 (29, 173)	164 (66, 283)	186 (110, –302)	55 (22, 184)

*Analysis only counted each patient’s first qualifying catheter insertion; ^†^Duration of catheter placement = time from catheter insertion to first qualifying outcome event, catheter replacement or removal, health plan termination, or study end.

CVC, central venous catheter; HD, hemodialysis; IQR, interquartile range; SD, standard deviation.

### Incidence of catheter-related complications

**Figure 2:**
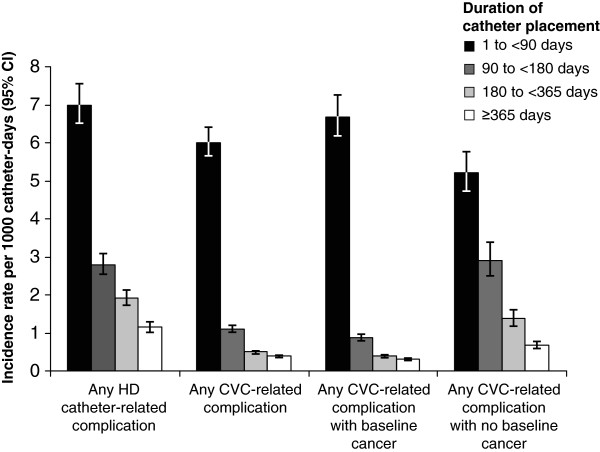
**Incidence of any catheter-related complication by baseline cancer status and duration of catheter placement.** Vertical bars indicate 95% confidence intervals (CIs). CVC, central venous catheter; HD, hemodialysis.

The IRs of any catheter-related complications by duration of catheter placement are depicted in Figure 2, categorized by patient cohort (HD catheter or CVC), as well as cancer-at-baseline status (CVC cohort only). CVC patients with cancer at baseline had statistically significantly higher rates of any complication compared with CVC patients without cancer during the first 90 days of catheter placement (*P* = .0001). For all catheter placement periods after 90 days, patients with cancer at baseline had statistically significantly lower complication rates than patients with no reported cancer in the same catheter placement period (Figure 2).

Table 2 presents the IRs of specific and any CVC and HD catheter–related complications among patients with catheter placement lasting less than 90 days. For specific complications, CRBSIs had the highest IR, 4.0 for CVCs (95% CI, 3.7-4.3) and 5.1 for HD catheters (95% CI, 4.7-5.6), followed by thrombosis, 1.3 for CVCs (95% CI, 1.1-1.4) and 0.8 for HD catheters (95% CI, 0.7-1.0). Other IRs for CVCs and HD catheters were lower, ranging from 0.09 (MB in CVCs) to 0.68 (MCRCs in HD catheters). The IRs for ICH were identical (0.10) in both cohorts. The incidences of CRBSIs, thromboses, and any complication were significantly higher in patients with cancer at baseline than in those with no reported cancer (*P* < .05 for each type of complication).

**Table 2:** Incidence rates of complications among patients with catheter placement^*^ **< 90 days**

**Type of complication**	**Number of complications per 1000 catheter-days (95% CI)**			
	**HD catheters**	**CVC all**	**CVC cancer**	**CVC no cancer**
	**n = 3213**	**n = 5060**	**n = 2479**	**n = 2581**
CRBSI	5.10	4.01	4.33^†^	3.62^†^
	(4.69-5.55)	(3.72-4.32)	(3.92-4.78)	(3.22-4.07)
Thrombosis	0.80	1.26	1.71^†^	0.76^†^
	(0.65-0.98)	(1.11-1.44)	(1.46-2.00)	(0.59-0.97)
MCRC	0.68	0.59	0.65	0.53
	(0.55 -0.85)	(0.49-0.72)	(0.50-0.83)	(0.40-0.72)
Embolism	0.49	0.39	0.38	0.40
	(0.38-0.64)	(0.31-0.49)	(0.27-0.52)	(0.28-0.56)
ICH	0.10	0.10	0.04^†^	0.17^†^
	(0.06-0.18)	(0.06-0.16)	(0.02-0.11)	(0.10-0.29)
MB	0.27	0.09	0.05	0.14
	(0.19-0.39)	(0.06-0.15)	(0.02-0.12)	(0.08-0.25)
Any^‡^	6.98	6.00	6.67^†^	5.20^†^
	(6.49-7.52)	(5.64-6.38)	(6.15-7.23)	(4.71-5.74)

*Analysis only counted each patient’s first qualifying catheter insertion; ^†^*P* < 0.05, Fisher’s exact test; ^‡^Defined as the initial occurrence of the first- or only-occurring complication.

CI, confidence interval; CRBSI, catheter-related bloodstream infection; CVC, central venous catheter; HD, hemodialysis; ICH, intracranial hemorrhage; MB, major bleeding; MCRC, mechanical catheter–related complication.

**Figure 3:**
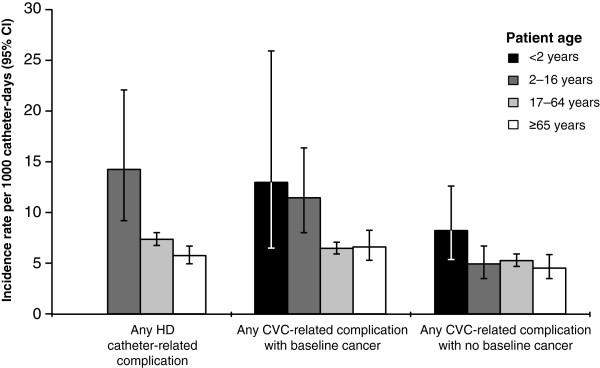
**Incidence of any catheter-related complication during the first 90 days of catheter placement.** Results are presented by patient cohort, patient age, and baseline cancer status. There were no children <2 years old in the HD cohort. Vertical bars indicate 95% confidence intervals (CIs). CVC, central venous catheter; HD, hemodialysis.

**Figure 4:**
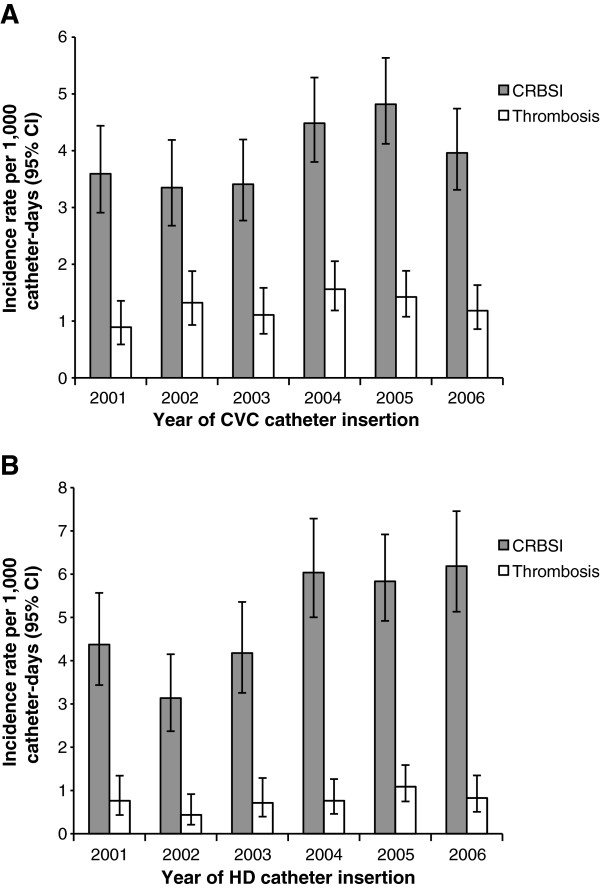
**Yearly incidence of CRBSI and thrombosis in patients with CVCs (A) or HD catheters (B).** Data for 2001 to 2006 are shown. Vertical bars indicate 95% confidence intervals (CIs). CRBSI, catheter-related bloodstream infection; CVC, central venous catheter; HD, hemodialysis.

The IRs of any catheter-related complications occurring in the first 90 days of catheter placement are shown by patient cohort, baseline cancer status, and patient age in Figure 3. Among CVC patients with baseline cancer, younger patients (in combined age groups <2 and 2–16 years, data not shown) had statistically significantly higher rates of any complication compared with older age groups (*P* = .0005). Complication rates did not differ between younger and older age groups in CVC patients without cancer. HD patients in age group 2–16 years had statistically significantly higher rates of complications versus older age groups (*P* = .0019). Temporal trends for the most frequent complications (i.e., CRBSI and thrombosis) were examined in both CVC and HD catheter cohorts over the 2001–2006 study period and there was no consistent evidence suggesting substantive changes in the incidence of either CRBSIs or catheter-related thrombosis during this period (Figure 4).

In the sensitivity analysis, during the first 90 days of catheter placement, the IRs for the most common complications decreased while rates for embolism, ICH, and MB increased slightly. The decrease in IR for CRBSI (from 4.0 to 1.6) was more substantial than for thrombosis (from 1.3 to 0.8) among patients in the CVC cohort (Additional file 2).

## Discussion

This study analyzed the incidence of catheter-related complications occurring in a real-world setting using a single, large population sample and consistent methods to identify and calculate unadjusted IRs for these different complications. The results demonstrate that the highest rates of first complications after CVC and HD catheterization occur during the first 90 days of catheter placement. Rates of complications are also higher in cancer patients with CVC placements than in non-cancer patients during the first 90 days of catheter insertion. Complication rates in children with HD catheters and with CVCs with cancer at baseline are higher than in the respective catheter cohorts for adults and elderly patients.

Earlier reports of IRs of catheter-related complications were characterized by great variability due to inconsistencies in terminology, study design and methodology, small sample sizes, and heterogeneity of patient populations. For example, some studies did not differentiate embolism from thrombosis [31], and others did not clearly define the specific types of “catheter malfunction” being studied [14,32,33]. This study utilized diagnostic and procedural codes from a large administrative claims database to extract data describing the most common complications following CVC or HD catheter insertion.

The finding that catheter-related complications were considerably more common during the first 90 days following catheter placement may reflect the fact that some catheter-related complications eventually lead to catheter removal (the censoring event in the present study). Recurrent catheter insertions and complications occurring after repeated catheter placements were not evaluated.

In this study, the most frequent complications associated with catheter placement were CRBSIs and catheter-related thrombosis. The incidence of CVC-related thrombosis was low overall (4.4% of catheters or 1.3/1000 catheter-days). In previous reports, the incidence of CVC-related thrombosis varied widely (0.6%-33% of catheters) because of differences in catheter type, study design and population, and sensitivity of the examination procedures [9]. There is growing evidence that catheter-related infection and thrombosis are closely related because both involve fibrin sheath formation, and the risk of clinically apparent thrombosis is markedly increased after an infection episode [9]. In part, this may account for these 2 types of complications having the highest IRs in both the CVC and HD catheter cohorts of our study.

A sensitivity analysis was also conducted to determine if eliminating the requirement for having a catheter removal or replacement claim had an effect on complication rates. Comparison of findings from the first 90 days of catheter placement (i.e., higher CRBSI IR with removal/replacement requirement) demonstrates that some complications, such as CRBSI, are more likely to result in catheter removal. While CRBSI typically presents with visible symptoms, up to two-thirds of patients with catheter-related thrombosis are asymptomatic [9,34,35]. In most instances, catheters causing CRBSI must be removed to resolve the problem [34]. In contrast, patients identified as having thrombosis can be treated with thrombolytic therapy to resolve the problem [35]. While patient age is considered a risk factor for CRBSI (CRBSI is more common in children than in adults [34]), the age distributions were similar in the main and sensitivity analyses. In the sensitivity analyses, IRs for relatively rare complications (e.g., embolism and ICH) slightly increased. These findings could be related to delayed effects of treatment of occluded catheters rather than catheter placement. Further studies are needed to investigate these findings.

Consistent with earlier reports, our study showed a higher frequency of catheter-related complications in patients with cancer than in non-cancer patients during the first 90 days of catheter placement, possibly because of the use of immunosuppressive cancer therapies. The risk of infection is known to be particularly high in neutropenic patients and in patients undergoing chemotherapy prior to stem cell transplantation [36]. More than one insertion attempt (OR, 5.5), ovarian cancer (OR, 4.8), and previous CVC insertion (OR, 3.8) are also significant baseline risk factors for catheter-related thrombosis in cancer patients [37]. In particular, the risk of developing CVC-related thrombosis is significantly higher in patients with cancer who also carry the factor V Leiden mutation (reported relative risk from 2.6 to 7.7) [38,39], or have hyperhomocysteinemia (reported ORs from 3.8 (95% CI, 1.3-11.3) [40] to as high as 33.9 (95% CI, 1.53-751.33) [41]), compared with patients without these conditions. Further analyses based on these and other risk factors are warranted, and may yield new insights into the management of cancer patients with indwelling catheters.

Although the incidence of catheter-related complications was significantly higher in CVC patients with cancer at baseline compared with non-cancer patients during the first 90 days of catheter placement, non-cancer patients (i.e., patients in HD catheter or CVC non-cancer groups) had greater incidences of any type of complication in catheter placement periods lasting 90 days or longer. One possible explanation for this finding is that over time, cancer patients (presumably the more severely ill group and inherently different from non-cancer patients) had poorer survival than the non-cancer patients, resulting in their reduced contribution to lengthier catheter time periods. It is also plausible that the severity of complications in these cancer patients necessitated earlier catheter removal (within the first 90 days), whereas in the non-cancer group, complications occurred and catheters were removed later. Because of limitations of the claims database, comorbid conditions and other factors possibly contributing to outcome events were not assessed in any of the groups.

This analysis was based on automated medical and prescription claims. While claims data are extremely valuable for the efficient and effective examination of health care outcomes, all claims databases have certain inherent limitations because the claims are collected primarily for the purpose of reimbursement for health services and not for research. The presence of a diagnosis code on a medical claim does not always indicate positive presence of disease because claims data are subject to errors in coding, inaccurate disease classification, or may include a 'rule out’ diagnostic workup rather than actual disease. To increase the specificity in outcome identification, we required 1 overnight hospitalization or a hospital emergency room visit and used validated diagnostic and procedural codes identifying these conditions from published literature [10-12,29,30]. Although we attempted to utilize precise codes to identify catheter type, cancer status, and outcome events, coding errors may have misclassified some patients. An additional potential confounder is the inability to distinguish from claims data whether catheterization was the cause of the defined outcome event or the indication for the outcome event. However, we imposed patient selection requirements (i.e., 180 days of outcome-free enrollment prior to index and record of catheter insertion *and* removal or replacement claims) in order to correctly ascertain the temporal relationship between the exposure and outcome event.

There is also a potential for misclassification regarding patient ascertainment to either HD catheter or CVC groups. However, to more accurately categorize patients in these 2 groups, we used coding recommended by the US Renal Data System and further required patients in the HD cohort not to have claims for chemotherapy or parenteral nutrition within 30 days of HD catheterization. Patients without HD procedure codes were allocated into CVC cohort.

Another limitation of our study was that patients in the database we utilized were commercially insured and might not be completely generalizable to the general US population. There is limited ability in claims data to determine whether demographic characteristics of the study population are similar to the general population. However, the age distribution of the study population was similar to that in the general US population during the same time period.

Previous studies showed that site of catheter insertion can influence the incidence of certain catheter-related adverse events such as infectious and thrombotic complications [22,42,43]. Limitations inherent to claims data did not allow us to determine the type of catheter device used (tunneled vs. non-tunneled devices), the site of the catheter insertion, or whether the catheter that was removed during the 90-day period was the same catheter that was placed. The extent to which “good” catheter placement and care recommendations are practiced can also influence complication rates; however, the claims data source did not permit us to determine whether techniques including sterilization methods (e.g., ethanol locks) were utilized at the clinical level. It was also not possible to determine whether lag time between the actual catheter removal or replacement and claims processing dates may have biased our results.

Future analyses examining the correlation of claims for infection and thrombosis may provide more insight into the possible simultaneous occurrence of these complications. While patients who require long-term vascular access are generally very sick, patients who require catheters for HD may differ in important ways from those who require CVADs for medical conditions other than hemodialysis. An understanding of these differences could provide further guidance for improving the care of patients who are severely or chronically ill.

## Conclusion

This study provides a new body of data on the risk of catheter-related complications derived from a large patient population. The risk of catheter-related complications is highest during the first 90 days of catheter placement in patients with CVC and HD catheters and in younger patients (≤16 years of age) with HD catheters. In younger patients (<2 years of age and 2–16 years of age, combined) with CVCs the risk of complications is higher than in patients over 16 years of age only in patients with cancer at baseline. This information provides valuable additional context for the development of strategies for the management of dysfunctional CVC and HD catheters and for improved patient care.

## Informed consent

Because it involved analysis of preexisting, de-identified data, this study was exempt from institutional review board approval.

## Abbreviations

CI: Confidence interval; CPT: Current procedural terminology; CRBSI: Catheter-related blood stream infection; CVAD: Central venous access device; CVC: Central venous catheter; HCPCS: Healthcare common procedure coding system; HD: Hemodialysis; ICD-9-CM: International classification of diseases, ninth revision, clinical modification; ICH: Intracranial hemorrhage; IR: Incidence rate; MB: Major bleeding; MCRC: Mechanical catheter–related complication; OR: Odds ratio.

## Competing interests

All authors are employees of Genentech, Inc.

## Authors’ contributions

PN, DF, and LC conceptualized and designed the project, performed the analysis, interpreted the data, and drafted the manuscript. All authors participated in critically revising the manuscript for important intellectual content and approved the final manuscript to be published.

## Pre-publication history

The pre-publication history for this paper can be accessed here:

http://www.biomedcentral.com/1471-2261/13/86/prepub

## Supplementary Material

Additional file 1 Central venous catheter (CVC) insertion CPT procedure codes and descriptions. Click here for file

Additional file 2 Incidence rates of complications among patients with catheter placement^* ^**< 90 days: comparison of results from main analysis and sensitivity analysis.** Click here for file
