# Supplementary material for: Incidence of catheter-related complications in patients with central venous or hemodialysis catheters: a health care claims database analysis
Source: BMC Cardiovasc Disord. 2013 Oct 16;13:86. doi: 10.1186/1471-2261-13-86 (PMC4015481; doi:10.1186/1471-2261-13-86)
Supplement: Additional file 2 — Incidence rates of complications among patients with catheter placement * < 90 days: comparison of results from main analysis and sensitivity analysis. [file 1471-2261-13-86-S2.doc]

**Appendix B.** Incidence rates of complications among patients with catheter placement^*^ < 90 days: comparison of results from main analysis and sensitivity analysis

| **Type of Complication** | **Number of Complications per 1,000 Catheter-Days (95% CI)** | | | | | |
| --- | --- | --- | --- | --- | --- | --- |
|  | **Main Analysis**** | | | **Sensitivity Analysis***** | | |
|  | **HD Catheters**  ***n* = 3,213** | **CVC All**  ***n* = 5,060** |  | | **HD Catheters**  ***n* = 9,631** | **CVC All**  ***n* = 25,681** |
| CRBSI | 5.10  (4.69-5.55) | 4.01  (3.72-4.32) |  | | 4.82  (4.58-5.07) | 1.56  (1.49-1.64) |
| Thrombosis | 0.80  (0.65-0.98) | 1.26  (1.11-1.44) |  | | 0.61  (0.54-0.70) | 0.79  (0.74-0.85) |
| MCRC | 0.68  (0.55 -0.85) | 0.59  (0.49-0.72) |  | | 0.10  (0.07 -0.14) | 0.07  (0.06-0.09) |
| Embolism | 0.49  (0.38-0.64) | 0.39  (0.31-0.49) |  | | 0.68  (0.60-0.77) | 0.80  (0.74-0.85) |
| ICH | 0.10  (0.06-0.18) | 0.10  (0.06-0.16) |  | | 0.22  (0.18-0.28) | 0.28  (0.25-0.32) |
| MB | 0.27  (0.19-0.39) | 0.09  (0.06-0.15) |  | | 0.19  (0.15-0.25) | 0.17  (0.15-0.20) |
| Any**** | 6.98 (6.49-7.52) | 6.00 (5.64-6.38) |  | | 6.55  (6.26-6.84) | 3.41  (3.30-3.53) |

*Analysis only counted each patient’s first qualifying catheter insertion; **Censoring in main analysis occurred at first qualifying outcome event, catheter replacement or removal, health plan termination, or study end; *** Censoring in sensitivity analysis occurred at first qualifying outcome event, health plan termination, or study end; ****Defined as the initial occurrence of the first- or only-occurring complication.

CI, confidence interval; CRBSI, catheter-related bloodstream infection; CVC, central venous catheter; HD, hemodialysis; ICH, intracranial hemorrhage; MB, major bleeding; MCRC, mechanical catheter‑related complication.
